# Indoor Air Quality in the Metro System in North Taiwan

**DOI:** 10.3390/ijerph13121200

**Published:** 2016-12-02

**Authors:** Ying-Yi Chen, Fung-Chang Sung, Mei-Lien Chen, I-Fang Mao, Chung-Yen Lu

**Affiliations:** 1 Institute of Environmental and Occupational Health Sciences, School of Medicine, National Yang Ming University, Taipei 11221, Taiwan; e23364@mail.trtc.com.tw; 2 Management Office for Health Data, China Medical University Hospital, Taichung 40402, Taiwan; fcsung1008@yahoo.com; 3 Department of Health Services Administration, College of Public Health, China Medical University, Taichung 40402, Taiwan; 4 Department of Occupational Safety and Health, Chung Shan Medical University, Taichung 40201, Taiwan; 5 Department of Medical Research, Chung Shan Medical University Hospital, Taichung 40201, Taiwan; 6 Department of Sport and Health Management, Da-Yeh University, No.168, University Rd., Dacun, Changhua 51591, Taiwan; 7 School of Post-Baccalaureate Chinese Medicine, China Medical University, Taichung 40402, Taiwan

**Keywords:** indoor air, subway, transit, particulate matter, metro

## Abstract

Indoor air pollution is an increasing health concern, especially in enclosed environments such as underground subway stations because of increased global usage by urban populations. This study measured the indoor air quality of underground platforms at 10 metro stations of the Taipei Rapid Transit system (TRTS) in Taiwan, including humidity, temperature, carbon monoxide (CO), carbon dioxide (CO_2_), formaldehyde (HCHO), total volatile organic compounds (TVOCs), ozone (O_3_), airborne particulate matter (PM_10_ and PM_2.5_), bacteria and fungi. Results showed that the CO_2_, CO and HCHO levels met the stipulated standards as regulated by Taiwan’s Indoor Air Quality Management Act (TIAQMA). However, elevated PM_10_ and PM_2.5_ levels were measured at most stations. TVOCs and bacterial concentrations at some stations measured in summer were higher than the regulated standards stipulated by Taiwan’s Environmental Protection Administration. Further studies should be conducted to reduce particulate matters, TVOCs and bacteria in the air of subway stations.

## 1. Introduction

In the metropolitan Taipei area of Taiwan, millions of people take the advantage of the convenience of the Taipei Rapid Transit system (TRTS), which substantially reduces traffic jams on city streets [1]. This system consists of five lines and 117 transfer stations with a 131.1-km length in the metro area [2], serving the majority of passengers from 06:00 to 24:00 on weekdays. There are three daily rush hours, from 7 to 9 a.m., from 5 to 7 p.m. and from 9 to 11 p.m. Most parts of the TRTS are underground, where various types of air pollutants, either generated internally or entering from the outside atmosphere, might be accumulated in a confined space. There is concern over whether the pollutants could pose a health risk to passengers and subway employees. With the fast-rising TRTS ridership, concerns about indoor air pollution have been raised. The indoor air pollutant control in the rapid transit system is important to commuters and employees. The Environmental Protection Administration (EPA) of Taiwan has considered that prolonged exposure to indoor air pollutants might affect job performance and could be detrimental to human health. The Administration thus implemented the Taiwan Indoor Air Quality Management Act (TIAQMA) to set maintenance limits for the main indoor pollutants including carbon dioxide (CO_2_), carbon monoxide (CO), formaldehyde (HCHO), total volatile organic compounds (TVOCs), ozone (O_3_), airborne particulate matters, including particles of 2.5–10 µm (PM_10_) and of ≤2.5 µm (PM_2.5_), bacteria and fungi [3,4] (Table 1).

The TIAQMA defines the scope of “Indoor” in the Act broadly, meaning “any closed or semi-closed space in buildings used by the public, and any mass transportation space that carries passengers”. However, the Taiwan EPA has decreased the number of regulated projects according to different categories and characteristics of the sites to reduce the cost of detection and the impact of industrial chemicals. The EPA requires that the indoor air quality (IAQ) of rapid transit systems to comply with the stipulated standards for CO, CO_2_, and HCHO [5]. Previous studies have associated poor indoor air quality with building-related symptoms and oxidative stress [6,7,8,9,10,11,12,13,14]. The accumulation of indoor CO_2_, HCHO, TVOCs, O_3_, PM_10_, bacteria and fungal contamination significantly increases the risks of sick building syndrome (SBS) [6,7,8,9,10,11], and high urinary 8-hydroxydeoxyguanosine (8-OHdG) levels [12,13,14]. Urinary 8-OHdG is considered as a biomarker for oxidative stress and has been significantly associated with building-related complaints in office workers [14,15], the exposure to polyaromatic hydrocarbons in traffic conductors and Chinese military cooks [16,17] as well as the exposure of PM_10_ and PM_2.5_ in bus drivers [18]. In addition, HCHO may cause nasal and eye irritation, neurological effects, and increased risk of asthma or allergies, and is genotoxic, causing DNA adduct formation and clastogenic effects [19]. It also has been classified as a group B1 carcinogen because of its carcinogenic potential to humans [20,21,22].

Many studies on indoor air quality in crowded stations of subways or metro systems have been conducted in many countries. High levels of PM_10_, PM_2.5_ and volatile organic compounds (VOCs) were reported for some subways or metro systems [23,24,25,26]. Johansson and Johansson reported that the mean PM_10_ and PM_2.5_ concentrations in the air of underground platforms of Stockholm’s subway system were 470 and 260 µg/m^3^, respectively, or more than 5 and 10 times higher than those of outdoor roads, respectively [23]. Aarnio et al. also reported that mean concentrations of PM_2.5_ in two subway stations of Helsinki’s subway system were 47 and 60 µg/m^3^, representing five to six times higher values than the mean outdoor concentration (19 µg/m^3^) [24]. A study of Tehran’s subway system found that the mean particulate matter levels in air samples from two indoor platforms were greater than that of outdoors for both PM_10_ (91.3 vs. 77.9 µg/m^3^) and PM_2.5_ (46.8 vs. 32.5 µg/m^3^) [25]. A recent study in Shanghai found elevated concentrations of VOCs in old metro carriages and carriages in urban areas because of poor air circulation and ventilation on the underground track. The measured average acetone and acrolein increased from 7.71 to 26.28 µg/m^3^ as the number of commuters increased from 40 to 200 people in the carriages [26].

Cheng et al. have measured PM_10_ and PM_2.5_ in the air of platforms at five TRTS stations for one hour and found the concentrations ranged from 11 to 137 µg/m^3^ and from 7 to 100 µm/m^3^, respectively [27,28]. The PM levels in the air of underground platforms were more than 10-fold greater than that of outdoor air. However, no other pollutants were measured. We, therefore, attempted to measure the 24-h indoor air quality at major TRTS stations to provide quantitative information on the temporal variations and spatial distributions for target indoor air pollutants. This study also intended to compare indoor air quality of the TRTS not only with the stipulated standards of CO, CO_2_, and HCHO for public transportation platforms, but also with other standards specified in the TIAQMA.

## 2. Materials and Methods

With the permit obtained from the Taipei Rapid Transit Corporation (TRTC), Taiwan, we identified for this study the 10 stations with the highest ridership among all TRTS stations. Indoor air samples were collected from and quantified for the underground platforms once in winter and once in summer. At these stations, the central heating ventilation and air-conditioning (HVAC) system set air changes per hour (ACH) at 4–10 changes regulated by the indoor and outdoor temperatures.

At each station, 24-h measures were performed for CO, CO_2_, humidity, temperature, O_3_, PM_10_ and PM_2.5_ on the platform at each station. A one-hour assessment for HCHO and TVOCs was also performed during the evening rush hour. A 5-min air sample of indoor airborne particles was also collected to evaluate the exposure to bacteria and fungi during the evening rush hour. All samples were collected at 1.5 m height and at least 0.5 m away from all surfaces of objects and ventilation systems to simulate the breathing zone of standing passengers. CO and CO_2_ were measured with the PPMonitor Stand Alone System (SAS) monitor (PPM Technology Ltd., Caernarfon, UK) with a detectable range of 0–100 ppm (resolution: 0.1 ppm), and a nondispersive infrared technique with a detectable range of 0–5000 ppm (resolution: 1 ppm), respectively. Calibrations were conducted using 2018 ppm CO_2_ and 100.4 ppm CO for the span gas as well as nitrogen for the zero gas before sampling. O_3_ and HCHO were measured with the PPMonitor SAS monitor with detectable ranges of 0–1 ppm (resolution: 0.01 ppm) and 0–10 ppm (resolution: 0.001 ppm), respectively. Calibrations were respectively conducted using 1.02 ppm O_3_ and 2.51 ppm HCHO for the span gas before sampling. Temperature and humidity were also measured with the PPMonitor SAS monitor by using a thermistor in a range from −40 °C to 128 °C (resolution: 0.01 °C) and an interchangeable capacitor with a detectable range of 0%–100% (resolution: 0.01%), respectively. TVOCs were measured with a photoionization detector (Model PGM-7240, RAE Systems, San Jose, CA, USA) with a range of 0–10,000 ppb (resolution: 1 ppb) and built-in correction for 102 built-in VOC gases. Calibration was conducted using 10,000 ppb isobutylene for the span gas and hydrocarbon-free air for the zero gas before sampling. The PM_10_ and PM_2.5_ levels were assessed using a portable dust monitor (Model TSI 8520, TSI Inc., Shoreview, MN, USA) by using a light scattering technique with a range of 0.001–100 mg/m^3^ (resolution: 0.001 mg/m^3^). Calibration was conducted using filter for zero count test before sampling. Bacteria and fungi were sampled using a bioaerosol impactor with a flow rate of 28.3 mL/min for 5 min. Calibration was conducted using a BioStage Single-stage Impactor (SKC Inc., Covington, GA, USA) with 28.3 L/min for flow test before and after sampling. After sampling, the samples were transported to the laboratory as soon as possible avoiding exposure to sunlight. For the quantitative analyses, samples were cultivated in a temperate box at 25 ± 1 °C for 5 days. The assessments of bacteria and fungi concentrations were performed by counting the number of colonies and then dividing by sampling air volume of 141.5 L. Table 2 summarizes the information of the instruments and operational conditions of the analyses. Data were set up by EXCEL version 14.0 (Microsoft Corp., Redmond, WA, USA) and analyzed by SPSS version 17.0 (SPSS, Inc., Chicago, IL, USA). The IAQ indices between seasons were compared using the *t*-test analysis with the significance level of 0.05. The correlations between IAQ indices and ridership were estimated using the Pearson’s correlation coefficient analysis.

## 3. Results

Monitoring for indoor air quality was conducted on the platforms at ten stations with the highest daily riderships in the TRTS (Table 3). Mean levels of CO_2_, CO, O_3_, TVOCs, HCHO, humidity, temperature, PM_10_ and PM_2.5_, bacteria and fungi were 578.2 ppm, 2.08 ppm, 0.01 ppm, 0.064 ppm, 0.013 ppm, 54.6%, 22.39 °C, 97.2 μg/m^3^, 75.4 μg/m^3^, 478.5 CFU/m^3^ and 269.0 CFU/m^3^ in winter, respectively, and 701.6 ppm, 2.26 ppm, 0.01 ppm, 0.738 ppm, 0.009 ppm, 66.4%, 26.61 °C, 80.9 μg/m^3^, 56.2 μg/m^3^, 995.2 CFU/m^3^ and 232.2 CFU/m^3^ in summer, respectively. The stations with the highest pollution levels varied by pollutant and season. They were CO_2_ at station F, TVOCs at station E, PM_2.5_ at station D and bacteria at station F measured in summer, and CO at station C, HCHO at station D, PM_10_ at station D and fungi at station H measured in winter. Humidity and temperature were significantly higher in summer than in winter.

**Figure 1:**
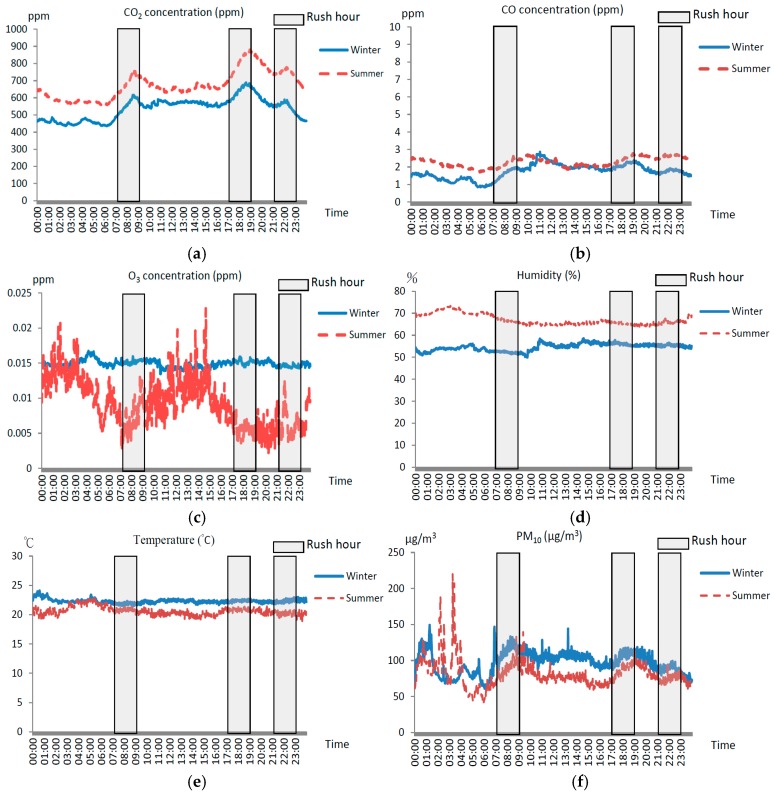

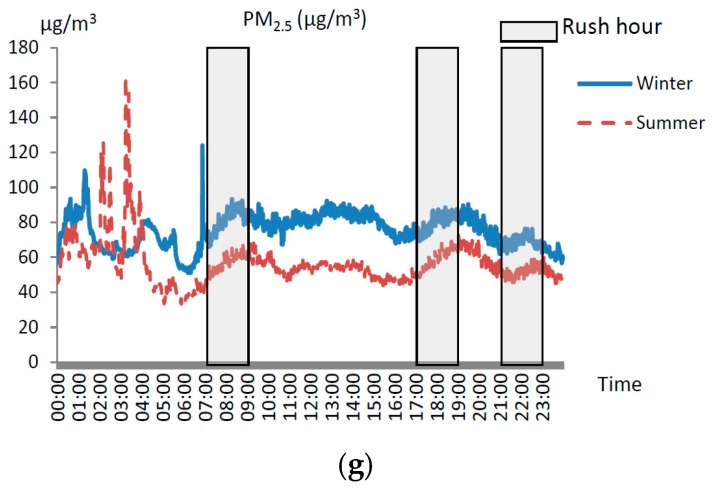
Mean hourly air quality measured from platforms of ten top most popular stations with top ridership in the Taipei Rapid Transit system (TRTS) in Taiwan. (**a**) CO_2_; (**b**) CO; (**c**) O_3_; (**d**) humidity; (**e**) temperature; (**f**) PM_10_; and (**g**) PM_2.5_.

Figure 1 shows the mean hourly levels of CO_2_, CO, O_3_, humidity, temperature, PM_10_ and PM_2.5_ for the 10 stations in the TRTS. There were three peak mean CO_2_ concentrations, 685 ppm at 8:34 a.m., of 776 ppm at 6:38 p.m. and of 680 ppm at 10:02 p.m. The hourly distribution trends were parallel in similar patterns in both seasons, but tended to be higher in summer than in winter. However, the difference had no statistical significance. Indoor CO concentrations remained at low levels in both seasons, ranging from 1.70 to 2.77 ppm. The mean O_3_ concentrations remained at a stable level of at about 15 ppb in winter. On the contrary, the O_3_ levels varied in summer and ranged from 2 ppb to 23 ppm, with two peak mean concentrations, 17 ppb at 0:08 a.m. and 23 ppb at 2:39 p.m.. The mean hourly indoor humidity mean levels in winter were obviously higher than those in summer, ranging from 63.8%–73.4% and from 49.9%–58.6%, respectively. Indoor mean temperature levels remained stable on a daily basis (18.9–24.1 °C), especially in winter (18.9–22.8 °C). The hourly trends of mean PM_10_ and PM_2.5_ concentrations were both parallel in winter and in summer, with the highest peaks appearing at midnight and increasing again during the morning rush hour. There was a peak of 219.9 μg/m^3^ PM_10_ at 3:17 a.m and a peak of 164.3 μg/m^3^ PM_2.5_ at 3:14 a.m. in Winter.

## 4. Discussion

Indoor CO, CO_2_, and HCHO levels in rapid transit systems are regulated by Taiwan’s EPA. However, we also compared the measured levels against all the air pollutant standards of the TIAQMA, including CO, CO_2_, HCHO, TVOCs, O_3_, PM_10_, PM_2.5_, bacteria and fungi. The 8-h measurements of CO_2_, CO and O_3_ levels for all stations met the standards. The measured 1-h level of HCHO was below the standard value of 0.08 ppm. The levels of pollutants except for ozone varied among stations and between seasons. The TVOCs levels were generally greater in summer than in winter. (Table 3). TVOC concentrations appeared highly variable across the stations, with mean levels that ranged from 0.016 to 0.137 ppm in winter and from 0.253 to 1.547 ppm in summer. For the five stations (A, D, E, H and J) in summer, TVOCs levels exceeded the indoor air quality standard of 0.56 ppm. The TVOCs at station E measured in summer was 97 times higher than that at station A measured in winter. However, further data analysis shows that TVOCs concentrations were not significantly associated with the ridership and CO_2_ concentrations, with Pearson’s correlation coefficients of 0.20 and 0.42, respectively. The reason for the increase of TVOCs concentration must be further investigated.

Bacteria concentrations exceeded the indoor air quality standard of 1500 CFU/m^3^ at three stations (D, E and F) in summer. Our further data analysis show that indoor bacterial concentrations were associated with the ridership and with indoor CO_2_ concentrations, with Pearson’s correlation coefficients of 0.58 and 0.76, respectively. The continued measurements in Figure 1 show that the CO_2_ mean levels among stations were closely associated with the ridership in both winter and summer, with a Pearson’s correlation coefficient of 0.66. It is likely that bacteria are carried into the indoor air and CO_2_ is generated by riders. The mean CO_2_ concentrations peaked during rush hours, but none of peak levels exceeded the TIAQMA standard limits. However, the average CO_2_ levels measured at stations E and F were near 1000 ppm because of the large amount of riders. This implied that the existing air conditioning and ventilation systems were unable to provide sufficient fresh air, resulting in the accumulation of pollutants.

CO in the air is generated mainly from incomplete combustion. In the TRTS, the average concentrations of CO remained at relatively low levels because there is no fossil fuel combustion source and smoking is prohibited in TRTS. The indoor O_3_ in the platforms may be released from indoor surface materials and enter from outdoor air. This study found that the average concentrations of O_3_ among stations were different between winter and summer. The difference could be associated with the indoor air change frequency, which was regulated by temperature sensors responding to the changes of indoor and outdoor temperatures. The O_3_ level decreased during rush hours in summer, which could be associated with low ACH and a larger number of commuters that increased the decay of O_3_. The Pearson’s correlation coefficient between O_3_ concentrations and ridership was −0.55.

Indoor humidity and temperature remained at relatively constant levels and were highly correlated with each other (Pearson’s correlation coefficient, 0.78) in this study. The humidity level was obviously higher in winter than in summer, reflecting the seasonal difference of the ambient atmosphere.

We found that the 24-h trends of PM_10_ and PM_2.5_ concentrations were parallel with same patterns in this study. However, it is important to note that most PM_10_ and PM_2.5_ measurements exceeded the 24-h indoor air quality standard levels of 75 and 35 µm/m^3^, respectively. It is also interesting that both the highest PM_10_ and PM_2.5_ levels appeared at midnight. These particular matters could be generated from the rail maintenance operations, including track inspection, and metallographic polishing and grinding, which produce aerosols. These aerosols could be re-suspended when the TRTS started the service in the early morning. Prevention and control of the indoor particulate matter releasing from the maintenance of rails at midnight should be implemented.

## 5. Conclusions

This study showed that among the indoor air pollutants measured at the 10 TRTS stations the concentrations of CO, CO_2_ and HCHO met the stipulated standards set by the TIAQMA. However, TVOCs levels measured at five stations and bacterial levels estimated at three stations in summer, and PM_10_ and PM_2.5_ concentrations exceeded the stipulated standards regulated by the EPA. The TRTS is a non-smoking system. The source of the increased TVOCs levels in the TRTS is unknown and should be further investigated. Indoor bacterial concentration in the TRTS was associated with the ridership and indoor CO_2_ concentrations. Increased air change rates in each station might reduce the exposure to bacteria and CO_2_. High PM_10_ and PM_2.5_ concentrations appeared at midnight, which could be associated with the maintenance of the railway tracks, and adequate control measures should be developed to reduce the generation of particulate matters.

## Figures and Tables

**Table 1:** The stipulated standards of Taiwan’s indoor air quality management act (TIAQMA).

Air Pollutant	Indoor Air Quality Standards
Carbon Dioxide (CO_2_)	1000 ppm (8-h Average)
Carbon Monoxide (CO)	9 ppm (8-h Average)
Formaldehyde (HCHO)	0.08 ppm (1-h Average)
Total Volatile Organic Compounds (TVOC)	0.56 ppm (1-h Average)
Bacteria	1500 CFU/m^3^ (Ceiling)
Fungi	1000 CFU/m^3^ (Ceiling)
particulate Matter of 2.5–10 µm (PM_10_)	75 μg/m^3^ (24-h Average)
particulate Matter of ≤2.5 µm (PM_2.5_)	35 μg/m^3^ (24-h Average)
Ozone (O_3_)	0.06 ppm (8-h Average)

**Table 2:** Instruments for indoor air quality assessment for the Taipei Rapid Transit System (TRTS).

Iterm	Equipment	Company	Analytical Principle	Detectable Range	Resolution	Calibration
CO	PPMonitor SAS monitor	PPM Technology Ltd., Caernarfon, UK	Non-Dispersion Infrared Detector	0–100 ppm	0.1 ppm	100.4 ppm
CO_2_	PPMonitor SAS Monitor	PPM Technology Ltd., Caernarfon, UK	Electrochemical	0–5000 ppm	1 ppm	2018 ppm
Humidity	PPMonitor SAS Monitor	PPM Technology Ltd., Caernarfon, UK	CMOSens Technology	0%–100%	0.01%	100%
Temperature	PPMonitor SAS Monitor	PPM Technology Ltd., Caernarfon, UK	CMOSens Technology	−40 °C–128 °C	0.01 °C	100 °C
O_3_	PPMonitor SAS Monitor	PPM Technology Ltd., Caernarfon, UK	Electrochemical	0–1 ppm	0.01 ppm	1.02 ppm
PM_10_	DUSTTRAK Aerosol Monitor 852	TSI Inc., Shoreview, MN, USA	Laser Photometer	0.001–100 mg/m^3^	0.001 mg/m^3^	0 mg/m^3^
PM_2.5_	DUSTTRAK Aerosol Monitor 852	TSI Inc., Shoreview, MN, USA	Laser Photometer	0.001–100 mg/m^3^	0.001 mg/m^3^	0 mg/m^3^
HCHO	PPMonitor SAS Monitor	PPM Technology Ltd., Caernarfon, UK	Electrochemical	0–10 ppm	0.001 ppm	2.51 ppm
TVOCs	RAE PGM-7240	RAE Systems, San Jose, CA, USA	Photoionization	0–10,000 ppb	1 ppb	10,000 ppb
Bacteria	SKC Biostage	SKC Inc., Covington, GA, USA	Impaction and Culture	-	-	-
Fungi	SKC Biostage	SKC Inc., Covington, GA, USA	Impaction and Culture	-	-	-

CO, carbon monoxide; CO_2_, carbon dioxide; O_3_, ozone; PM, particulate matter; HCHO, formaldehyde; TVOCs, total volatile organic compounds.

**Table 3:** Indoor air quality of the platforms of ten stations with top ridership in Taipei Rapid Transit system in Taiwan.

Station	Season	CO_2_ (ppm)	CO (ppm)	O_3_ (ppm)	TVOCs (ppm)	HCHO (ppm)	Humidity (%)	Temperature (°C)	PM_10_ (μg/m^3^)	PM_2.5_ (μg/m^3^)	Bacteria (CFU/m^3^)	Fungi (CFU/m^3^)	Riders (Number)
Standard ^a^	-	1000 ^a^	9 ^a^	0.06 ^a^	0.56 ^a^	0.08 ^a^	-	-	75 ^a^	35 ^a^	1500 ^a^	1000 ^a^	-
A	Winter	494	1.9	0.01	0.016	0.006	41	20.6	96 ^b^	75 ^b^	280	250	96,355
	Summer	679	3.0	<0.01	1.439 ^b^	0.015	63	25.6	102 ^b^	58 ^b^	201	363	95,808
B	Winter	427	2.3	0.01	0.026	<0.001	40	21.5	147 ^b^	101 ^b^	150	320	39,283
	Summer	589	2.4	0.01	0.253	0.004	65	26.9	135 ^b^	134 ^b^	193	123	37,577
C	Winter	641	2.7	0.01	0.072	0.011	59	23.7	128 ^b^	108 ^b^	220	205	47,763
	Summer	660	2.0	0.01	0.288	0.001	65	26.8	66	44 ^b^	385	122	49,099
D	Winter	648	3.5	0.02	0.109	0.042	61	21.5	111 ^b^	88 ^b^	660	135	47,700
	Summer	657	2.5	0.01	1.338 ^b^	0.015	66	25.2	103 ^b^	60 ^b^	1801 ^b^	312	45,320
E	Winter	760	1.2	0.01	0.064	0.010	55	21.9	55	39 ^b^	1335	205	110,126
	Summer	906	2.6	0.01	1.547 ^b^	0.015	64	26.7	91 ^b^	56 ^b^	2693 ^b^	189	135,394
F	Winter	663	1.4	0.01	0.049	0.008	56	21.3	63	45 ^b^	480	160	136,167
	Summer	919	2.4	<0.01	0.392	0.003	66	27.3	99 ^b^	75 ^b^	3253 ^b^	192	143,548
G	Winter	493	2.8	0.02	0.044	0.001	59	23.4	77 ^b^	59 ^b^	360	325	66,079
	Summer	651	2.0	0.01	0.290	0.008	71	26.9	49	37 ^b^	283	157	68,089
H	Winter	555	2.2	0.01	0.063	0.007	59	23	101 ^b^	80 ^b^	380	655	87,205
	Summer	661	2.0	0.01	0.811 ^b^	0.008	71	26.8	101 ^b^	60 ^b^	227	455	92,299
I	Winter	570	1.7	0.02	0.134	0.019	56	23.8	103 ^b^	75 ^b^	715	135	77,064
	Summer	840	2.7	0.01	0.384	0.004	63	28.1	40	27	737	300	79,385
J	Winter	531	1.1	0.01	0.066	0.011	60	23.2	91 ^b^	84 ^b^	205	300	13,100
	Summer	454	1.0	0.01	0.642^b^	0.015	70	25.8	23	11	179	109	13,032
Mean (S.D.)	Winter	578.2 (99.5)	2.08 (0.77)	0.01 (0.01)	0.064 (0.035)	0.013 (0.012)	54.6 (7.7)	22.39 (1.15)	97.2 ^b^ (28.0)	75.4 ^b^ (22.3)	478.5 (355.2)	269.0 (153.5)	72,084.2 (36,777.9)
	Summer	701.6 (145.5)	2.26 (0.55)	0.01 (<0.01)	0.738 ^b^ (0.517)	0.009 (0.006)	66.4 (3.1)	26.61 (0.86)	80.9 ^b^ (34.9)	56.2 ^b^ (33.1)	995.2 (1159.8)	232.2 (118.4)	75,955.1 (42,069.6)
*p*-Value ^c^		0.06	0.81	0.08	<0.01 ^d^	0.56	<0.01 ^d^	<0.01 ^d^	0.27	0.18	0.23	0.61	0.88

^a^ The stipulated standards of Taiwan’s indoor air quality management act (TIAQMA); ^b^ Measurement exceeded stipulated standards of Taiwan’s indoor air quality management act (TIAQMA); ^c^ Probability associated with a Student‘s *t*-Test, with a two-tailed distribution; ^d^ Statistically significant.
